# Hemolytic reaction in the washed salvaged blood of a patient with paroxysmal nocturnal hemoglobinuria

**DOI:** 10.1186/s12871-019-0752-4

**Published:** 2019-05-22

**Authors:** Yuko Kawamoto, Tasuku Nishihara, Aisa Watanabe, Kazuo Nakanishi, Taisuke Hamada, Amane Konishi, Naoki Abe, Sakiko Kitamura, Keizo Ikemune, Yuichiro Toda, Toshihiro Yorozuya

**Affiliations:** 1 Department of Anesthesia and Perioperative Medicine, Ehime University Graduate School of Medicine, Shitsukawa, Toon, Ehime 791-0295 Japan; 2 Department of Anesthesiology, Ehime Prefectural Imabari Hospital, Imabari, Ehime Japan; 3 Department of Anesthesiology and Intensive Care Medicine, Kawasaki Medical School, Kurashiki, Okayama, Japan

**Keywords:** Paroxysmal nocturnal hemoglobinuria, Blood salvage device, Autologous blood transfusion, Complement, Hemolysis, Hyperkalemia, Potassium

## Abstract

**Background:**

In patients with paroxysmal nocturnal hemoglobinuria (PNH), the membrane-attack complex (MAC) formed on red blood cells (RBCs) causes hemolysis due to the patient’s own activated complement system by an infection, inflammation, or surgical stress. The efficacy of transfusion therapy for patients with PNH has been documented, but no studies have focused on the perioperative use of salvaged autologous blood in patients with PNH.

**Case presentation:**

A 71-year-old man underwent total hip replacement surgery. An autologous blood salvage device was put in place due to the large bleeding volume and the existence of an irregular antibody. The potassium concentration in the transfer bag of salvaged RBCs after the wash process was high at 6.2 mmol/L, although the washing generally removes > 90% of the potassium from the blood. This may have been caused by continued hemolysis even after the wash process. Once activated, the complement in patients with PNH forms the MAC on the RBCs, and the hemolytic reaction may not be stopped even with RBC washing.

**Conclusions:**

Packed RBCs, instead of salvaged autologous RBCs, should be used for transfusions in patients with PNH. The use of salvaged autologous RBCs in patients with PNH should be limited to critical situations, such as massive bleeding. Physicians should note that the hemolytic reaction may be present inside the transfer bag even after the wash process.

## Background

Paroxysmal nocturnal hemoglobinuria (PNH; OMIM 300818) is a rare disease, which is caused by a mutation in the *PIGA* gene on chromosome Xp22 [1]. Rare Disease Database of National Organization for Rare Disorders reports that the estimated prevalence is 0.5–1.5 per million people in the general population. The mutation in the *PIGA* gene causes the deficit or lack of glycosylphosphatidylinositol (GPI)-anchored proteins, and as a result, GPI-anchored type factors regulating the complement system on the membrane of red blood cell [CD55 or decay-accelerating factor (DAF) and CD59] are deficient. Therefore, red blood cells (RBCs) are destroyed by the membrane-attack complex (MAC) formed by the body’s own activated complement system.

The use of transfusion therapy for patients with PNH has been well studied. Washed RBCs lacking white blood cells and complement components were used in the past, but the practice of washing the RBCs was deemed unnecessary and packed RBCs are usable without any problem for patients with PNH [2]. However, no reports have focused on the use of perioperative salvaged autologous RBCs for patients with PNH. In here, we describe a case involving the use of salvaged autologous RBCs in a patient with PNH.

## Case presentation

A 71-year-old man was scheduled to undergo total hip replacement surgery under general anesthesia to fix malunion of the right hip joint. Two months before the scheduled procedure, he had undergone left bipolar hip arthroplasty and right acetabular fracture fixation due to bilateral acetabular cartridge fractures. After the fractures, the patient had been prescribed oral polystyrene sulfonate calcium because of hyperkalemia. He was diagnosed to have PNH at the age of 60, and the oral administration of prednisolone was initiated. The therapy with eculizumab was not initiated.

### Preoperative examination

The preoperative blood examination showed pancytopenia [white blood cells, 2.100/μl; hemoglobin (Hb), 12.7 g/dl; and platelets, 100 × 10^3^/μl]. We suspected a hemolytic reaction due to the presence of a slightly increased aspartate aminotransferase, although bilirubin and lactase dehydrogenase level were within the normal limits. The hyperkalemia improved with the polystyrene sulfonate calcium. The irregular antibody screening was positive. Therefore, 6 units of packed RBCs and a blood salvage device (electa™; Sorin Group Italia, Italy) were prepared. No other abnormal results in the cardiac, liver, or renal functions were observed.

### Intraoperative findings

**Fig. 1:**
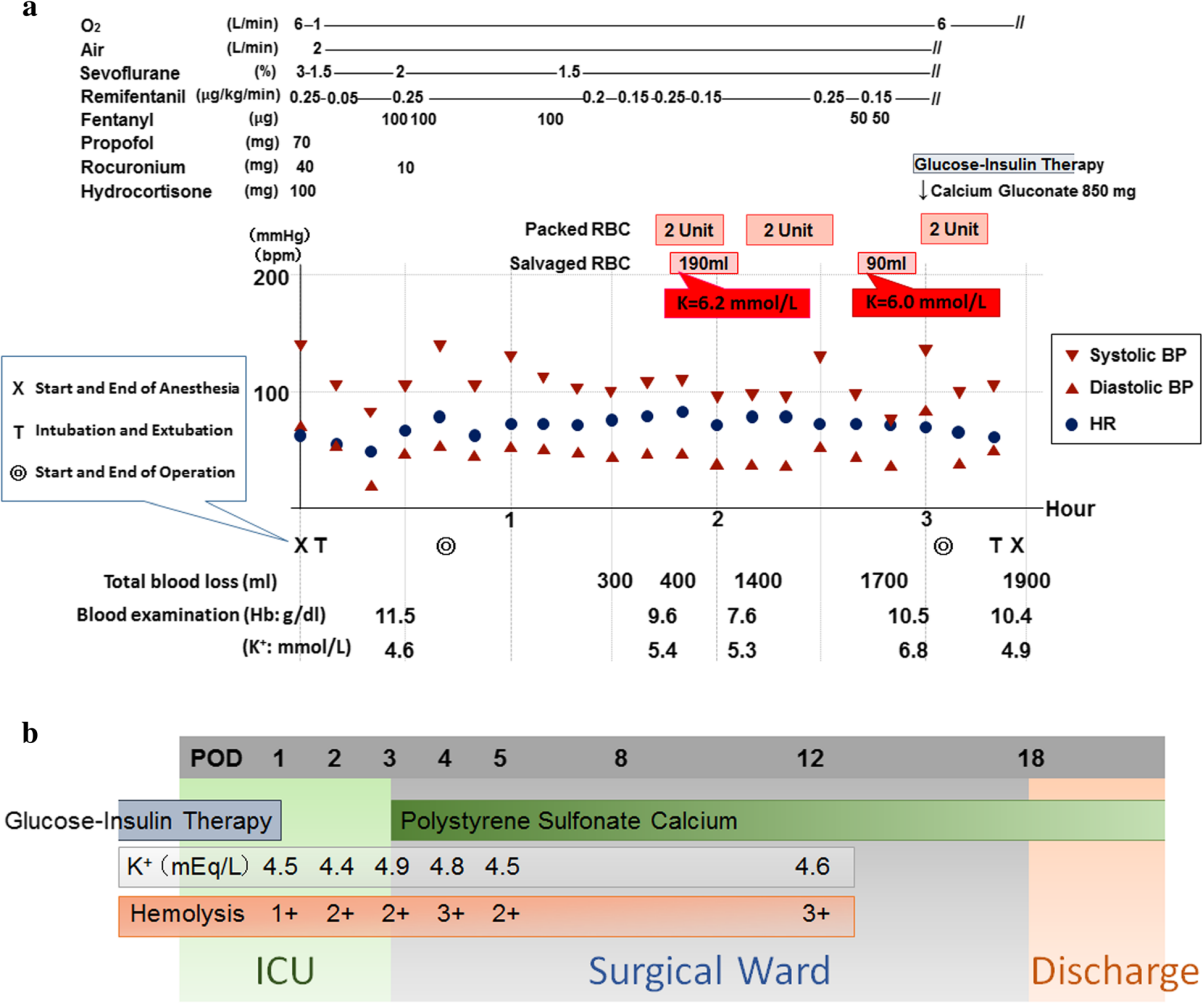
**a** Intraoperative progress. **b** Postoperative progress

Figure 1a depicts the intraoperative progress course. The Hb and potassium (K^+^) levels after the anesthesia induction were 11.5 g/dL and 4.6 mmol/L, respectively. An hour after the operation started, the same levels became 9.6 g/dL and 5.4 mmol/L, respectively, due to unexpected bleeding and presumably intravascular hemolysis. We initiated blood salvage procedures and started transfusion of 2 units of prepared packed RBCs using a potassium adsorption filter. After that, 190 ml of the first salvaged autologous RBCs were re-infused. Blood examination results to check K^+^ concentration levels in the transfer bag showed a high level of 6.2 mmol/L in the salvaged RBCs. Because the patient’s Hb became 7.6 g/dL due to continuous bleeding, we transfused two more units of packed RBCs and re-infused 90 ml of the second salvaged autologous RBCs using a potassium adsorption filter. The value of K^+^ in the transfer bag of the second salvaged autologous RBCs batch was also high at 6.0 mmol/L. The patient’s Hb level recovered to 10.5 g/dL after the RBC transfusion. However, the hyperkalemia progressed to 6.8 mmol/L of K^+^, and we administered 850 mg of calcium gluconate and initiated glucose-insulin therapy. Although the operation was close to being finished, we transfused two more units of packed RBCs anticipating the possibility of intravascular hemolysis after the operation. The surgery was performed without complications. The value of K^+^ at the end of the operation was 4.9 mmol/L. The amount of bleeding during the operation was 1900 ml, and the infusion volume during the operation was 2400 ml of crystalloids, 6 units of packed RBCs, and 280 ml of salvaged autologous RBCs. The duration of surgery and anesthesia was 140 and 215 min, respectively. Postoperatively, the patient was transferred to the intensive care unit (ICU).

### Postoperative course

Figure 1b indicates the patient’s progress after the operation. Glucose-insulin therapy was continued until the postoperative day (POD) 1. The patient left the ICU and restarted the oral intake of polystyrene sulfonate calcium at POD 3 because the K^+^ increased again. At POD 18, the patient was transferred to another hospital for rehabilitation.

## Discussion and conclusion

In normal blood, hemolytic reactions do not occur in the absence of antigen-antibody reactions, but the hemolytic reactions in patients with PNH are independent of antigen-antibody reactions. In patients with PNH, RBCs are destroyed by an activated complement system due to the deficit of membrane proteins DAF and CD59, which inhibit the formation of the MAC.

Transfusions in patients with PNH used to be performed with saline washed RBCs to reduce the risk of leukocyte sensitization, antibody production against human leukocyte antigens, and reactions which may activate complement system [3]. However, a review on the subject concluded that the use of washed RBCs is not necessary and group-specific fresh blood and blood products should be used instead [2, 4]. For our patient, we prepared 6 units of packed RBCs and an autologous blood salvage device because of the positive irregular antibody screening result. Autologous blood transfusion can avoid risks or side effects associated with blood transfusions. Moreover, the autologous blood salvage device can eliminate over 90% of plasma components [5] presumably including complement factors, which (in theory) would be an added advantage for patients with PNH, although the RBCs themselves are still vulnerable. In the past, over 90% of the K^+^ in salvaged blood was shown to be removed by the wash process [the washed RBCs were left with 1–2 mmol/L of (K^+^)] [6], but in our case, the K^+^ concentration in the transfer bag was > 6 mmol/L. This fact indicates that the salvaged blood of patients with PNH continued to be lysed even after the wash process. Physical stresses, such as infections and surgery, can cause complement activation [7]. In addition, the unwashed salvaged blood contains increased levels of proinflammatory cytokines, such as interleukin-1β, 6, and 8, and activated complement components compared to the circulatory blood [8, 9]. We suspect the hemolysis in our patient was caused by surgical stress or surgical inflammation as the K^+^ concentration increased gradually during the operation. The attack to the RBCs by the activated complement may have proceeded also in the reservoir of blood salvage device. The level of activated proinflammatory cytokines and activated complement in the salvaged blood is reduced by the wash [8]; however, once the complement in the patients with PNH becomes activated and starts forming MAC on the RBCs during the processes of the operation and blood salvage, the hemolytic reaction may not be stopped even with the washing procedure. That is, the MAC on the membrane of RBCs does not get washed off. Recently, eculizumab, a monoclonal antibody to the complement component 5 (C5), is widely used in PNH therapy. However, not all the patients with PNH are administered eculizumab because of several reasons such as indication, insurance, and patients’ choice. Eculizumab inhibits C5, resulting in the inhibition of MAC formation and the effect of eculizumab is remarkable [1, 10, 11]. Although further reports are needed, if eculizumab had been used in the present case, the results might have been different.

Based on our experience, packed RBCs using a potassium adsorption filter, if available, should be preferred to autologous blood in case of perioperative transfusion in patients with PNH. Kathirvel S et al. has mentioned that in general unwashed RBCs can be used without problems, but for large blood transfusion volumes, the RBCs may need to be washed [12]. Some anesthesiologists may consider the use of washed autologous RBCs under some situations, such as massive hemorrhage or existence of irregular antibodies; however, a pitfall lurks in the autologous RBCs of the patients with PNH. The washed autologous RBCs may transiently guarantee oxygen transport to the tissues, but the salvaged RBCs in patients with PNH will become the target of the complement and will get lysed again after the transfusion.

Conclusively, packed RBCs instead of salvaged autologous RBCs should be used for transfusions in patients with PNH. The use of salvaged autologous RBCs in patients with PNH should be limited to critical situations such as massive bleeding and physicians should keep in mind that the hemolytic reaction may be present inside the transfer bag even after the wash process.

## Abbreviations

PNH: Paroxysmal nocturnal hemoglobinuria
GPI: Glycosylphosphatidylinositol
DAF: Decay-accelerating factor
RBCs: Red blood cells
MAC: Membrane-attack complex
K^+^: Potassium
ICU: Intensive care unit
POD: Postoperative day
